# Anti-CD20 Treatment of Autoimmune Hemolytic Anemia Refractory to Corticosteroids and Azathioprine: A Pediatric Case Report and Mini Review

**DOI:** 10.1155/2018/8471073

**Published:** 2018-08-26

**Authors:** Alexandros Makis, Zoi Kanta, Dimitrios Kalogeropoulos, Nikoloaos Chaliasos

**Affiliations:** Department of Pediatrics, University Hospital of Ioannina, Ioannina, Greece

## Abstract

Autoimmune hemolytic anemia (AIHA) is a relatively uncommon hematological entity in children and sometimes is characterized by a severe course requiring more than one line course therapy. Treatment decisions depend on the severity and chronicity of the anemia and the characteristics of the autoantibodies. Immunosuppression with corticosteroids is the first-line treatment, especially in warm-reactive AIHA. Refractory cases are treated with immunosuppressive drugs, cytotoxic agents, androgens, or splenectomy, with various side effects and questionable efficacy. Another second-line option is rituximab, an anti-CD20 monoclonal antibody, which has been used as an off-label agent with encouraging results from small limited studies or case reports. Herein, we add our experience on the safety and clinical efficacy of rituximab by presenting the case of a boy with warm-type AIHA resistant to corticosteroids and azathioprine, successfully treated with rituximab. We also offer a review of the relevant literature.

## 1. Introduction

Autoimmune hemolytic anemia (AIHA) is rare in children, and it is characterized by the presence of autoantibodies (IgM and IgG) against erythrocyte membrane antigens, leading to their premature elimination by the macrophages of the reticuloendothelial system. AIHA is determined as primary or secondary depending on the presence of an underlying systemic illness, such as infections, autoimmune diseases, immunodeficiencies, malignancies, and drug exposure. Primary AIHA is subdivided in warm-reactive AIHA, paroxysmal cold hemoglobinuria, and cold agglutinin disease, according the type of antibody, its optimal binding temperature, and the fixation of the complement [1, 2].

Warm-type AIHA is the most common type accounting approximately for half of the pediatric cases. Warm-reactive autoantibodies, primarily IgG, attach on erythrocyte membrane antigens at 37°C, sometimes fix the complement, and lead to Fc receptor-mediated clearance by the macrophages in the spleen and other parts of the reticuloendothelial system [3]. Children present with anemia, jaundice, and mild splenomegaly. In most cases of warm-type AIHA, transfusions must be avoided due to the risk of further hemolysis, and the administration of corticosteroids, mainly prednisolone, is the best first-line option. However, if the anemia is severe (hemoglobin below 5 g/dL or is rapidly dropping), cardiovascular compromise might occur and erythrocyte transfusions are needed [4, 5].

In a significant proportion of children with warm-type AIHA (20%), the disease can have a chronic course with resistance or dependency to corticosteroids with subsequent side effects, requiring the use of second-line choices which have not been clearly established in children [4, 6]. More specifically, splenectomy and immunosuppressive drugs such as azathioprine, cyclophosphamide, cyclosporine, mycophenolate mofetil, or immunomodulating agents have been used as sole or combined options.

Rituximab, a chimeric monoclonal antibody targeting the CD20 antigen on B lymphocytes, has emerged as an off-label option in order to avoid splenectomy and the serious complications of corticosteroids and immunosuppressive drugs [7, 8]. In adults, a recent meta-analysis from several studies showed that the overall response rate and complete response rate were nearly 70% and 40%, respectively, for warm AIHA [9]. In children, the majority of the available publications are case reports or single-center uncontrolled studies [4, 7, 8, 10–14]. In a recent prospective national French study of 61 children, the 6-year relapse-free survival was 48% [15].

The purpose of this paper is to describe the case of a young boy with warm-reactive AIHA, refractory to corticosteroids, and azathioprine, who exhibited late response to rituximab and to present the relevant literature.

## 2. Case Report

A previously healthy 3.5-year-old male was referred to our department because of a five-day history of weakness and pallor. Two weeks before, a three-day history of diarrhea was mentioned. The past medical history is unremarkable, and no family history of hematological problems or autoimmune disorders was reported.

Physical examination revealed jaundice, pallor, and splenomegaly. The boy's heart rate was 125 beats/min, and a 2/6 systolic heart murmur was present. The initial laboratory investigation showed: hemoglobin 5.1 g/dL, absolute reticulocyte count 220 × 10^3^/*μ*L, mean corpuscular volume 75 fl, white blood cells 9.3 × 10^9^/L, and platelets 255 × 10^9^/L. The peripheral blood film showed polychromasia and spherocytes without schistocytes. Serum lactate dehydrogenase levels were 1540 U/L, total bilirubin 3.8 mg/dl, and indirect bilirubin 0.9 mg/dl. The renal and liver function tests showed calcium and phosphate were normal. Urine examination was negative for hemoglobin and myoglobin. The direct antiglobulin test (DAT) was strongly positive for IgG autoantibodies with no fixation of the complement. The results of antinuclear antibody and anti-deoxyribonucleic acid were negative. Serum C3 and C4 as well as IgG, IgM, and IgA levels were normal. Serology for cytomegalovirus, Epstein–Barr virus, *Mycoplasma pneumonia,* and human immunodeficiency virus were negative. Based on the symptoms, the clinical findings and the laboratory tests the warm type of AIHA was established.

Erythrocyte transfusion was firstly administered because of the severe anemia in order to avoid cardiovascular compromise. Intravenous methylprednisolone was also initiated at a dose of 3 mg/kg/day for the first 72 hours with excellent hematological response. When the boy was clinically stable, oral prednisolone at a dose of 2 mg/kg/day was then used for 4 weeks followed by a slow taper during the following 5 months. At that time, prednisolone was discontinued, and a relapse occurred after an upper respiratory infection as shown in Table 1.

For this reason, prednisolone was restarted at 2 mg/kg/day with a good hematological response. Due to the steroids side effects, the dose of prednisolone was slightly reduced two weeks later and a hematological deterioration occurred. After that, the dose was increased again to 2 mg/kg for 1-month period. The expected side effects, such as blood pressure above the 95th percentile, bilateral posterior subcapsular cataract, facial edema, and hairy back, were quite evident, and it was decided to slowly discontinue prednisolone and to administer azathioprine at a dose of 2 mg/kg/day.

Azathioprine was given for 4 months with a slight hematological deterioration despite the increase up to 3 mg/kg/day, as shown in Table 1, while the boy suffered from mumps, possibly due to the underlying immunosuppression. At this point, rituximab was initiated after informed written consent. The agent was given in 4 weekly doses at a dose of 375 mg/m^2^, with no infusion-related side effects. Stable response was noticed 2 months after initiation of rituximab (Table 1). Immunoglobulin levels remained normal without the need for replacement therapy with intravenous immunoglobulin (IVIG). The DAT became negative 12 months later. The boy remains disease-free without relapses, 3 years after the first presentation.

## 3. Discussion

In our report, we describe the successful use of rituximab in a child with warm-type AIHA, priorly treated with corticosteroids and azathioprine. Rituximab was a valuable option that allowed corticosteroid and azathioprine discontinuation due to unresponsiveness and side effects.

Corticosteroids represent the most effective first-line treatment for warm AIHA and especially for the IgG-related type. Their action is associated with a decrease in hemolysis by blocking the mononuclear phagocytic Fc gamma receptor leading to a rapid response within 24 to 48 hours. They also have a late negative effect in the autoantibody production, but this demands several weeks [16, 17]. In cases with severe anemia, intravenous methylprednisolone is usually administered at a total dose of 1 to 2 mg/kg/day for the first 1 to 3 days. After the initial stabilization, a scheme of oral prednisone at a dose of 1 to 2 mg/kg/day is then preferred for two to four weeks, followed by a slow discontinuation over two to six months, with a good overall response rate of approximately 80 percent [1, 5, 18]. However, in some cases AIHA can be resistant and dependent to large doses of corticosteroids or to long-term therapy resulting in severe and adverse systemic effects, as was the case in our patient [17, 19].

In order to avoid the corticosteroid side effects, splenectomy or administration of immunomodulatory drugs can be used. However, these therapies are not consistently effective and present no negligible risk of infectious complications due to their profound immunosuppression.

Splenectomy is the surgical removal of the main site of erythrocyte destruction and autoantibody production. It is considered in children above the age of five, because in younger ages there is a significant risk of postsplenectomy sepsis caused by encapsulated bacterial organisms. Children with IgG-derived AIHA have a good response up to 65% [5, 20, 21]. Despite that, splenectomy as rarely an option, because several second-line drugs exist in order to avoid the long-term risks.

Several immunomodulatory agents are available for children with refractory disease, such cyclosporine, mycophenolate mofetil, azathioprine, rapamycin, danazol, cyclophosphamide, vincristine, vinblastine, and eculizumab. Each of these agents is less effective than either corticosteroids or splenectomy. Cyclosporine, mycophenolate mofetil, azathioprine, and rapamycin act by targeting T-lymphocytes, cause immunosuppression, and have several hematological and renal side effects [22–24]. Clinical response may require several weeks, as happened in our case who also suffered from mumps due to the severe immunosuppression. In a recent study, rapamycin has been shown to be effective and safe in children with multiresistant AIHA [25]. Danazol is a synthetic androgen with multiple mechanisms of action which decreases autoantibody production and complement activation. Among their widely known implications, they affect hepatic transaminases and cause elevation of masculinizing hormones [26]. Cyclophosphamide, vincristine, and vinblastine are cytotoxic agents that diminish the autoantibody production. Limitation of their use in children is the myelosuppressive and mutagenic effects [27]. Eculizumab is a monoclonal antibody targeting C5 complement that has been recently shown to be effective in complement-mediated AIHA [28, 29].

Therefore, there is a great need for the introduction of new effective and safer pharmaceutical options. The anti-CD20 monoclonal antibody rituximab has been successfully used to treat children with refractory AIHA. The preferred scheme is four weekly intravenous infusions (375 mg/m^2^/dose). During the last years, several reports have emerged, along with few single-center uncontrolled studies and one very recent prospective study [4, 7, 8, 10–15]. The basic characteristics of these case reports and studies are presented in Table 2. As it is apparent, rituximab is a valuable option in refractory primary AIHA as well as in secondary cases, such as after bone marrow transplantation, related to primary immunodeficiencies and Evan's syndrome. Although hematological responses are promising, secondary hypogammaglobulinemia and prolonged B lymphopenia will occur after rituximab therapy. Close monitoring of immunoglobulin levels is necessary. Therefore, in patients who present with severe or recurrent infections and concomitant low immunoglobulin levels, intravenous immunoglobulin (IVIG) may be useful as replacement therapy after rituximab use. In a few cases, IVIG supplementation was administrated for six months after rituximab treatment until the hypogammaglobulinemia resolved [30]. Generally, rituximab is considered a well-tolerated treatment as only a small number of children from the literature needed IVIG and prophylaxis for possible infectious complications [4, 15].

Very recently, a prospective national observational study was published by Ducassou et al. on the benefits of rituximab as a second-line treatment for AIHA in children [15]. For the first time in the literature, the investigators estimate the clinical value of rituximab by achieving a four-year median follow-up. The response was better in primary, isolated AIHA and in infants when used early in the course of treatment. Rituximab allowed steroid tapering in 60–70% of patients with 30% showing long-term response. Regarding safety, allergic reactions were rare and no case of progressive multifocal leucoencephalopathy was observed. The major safety problems were prolonged hypogammaglobulinaemia in few cases and severe neutropenia in one case. Therefore, it seems rational to propose rituximab as a second-line therapy, if response is not observed with first-line corticosteroids.

In conclusion, corticosteroids remain the cornerstone of the first-line therapeutic treatment for AIHA. Nevertheless, according to the existing literature data, the implementation of rituximab as a second-line therapy is relatively safe and can resolve difficult cases of AIHA resistant to corticosteroids. Therefore, physicians who treat children with AIHA should be aware of this rational therapeutic tool, even from the early stages of nonresponsive cases, in order to avoid other aggressive regimens such as splenectomy or immunomodulation.

## Figures and Tables

**Table 1:** Hematological parameters and treatment during the course of the disease.

	Admission	4th day	4th week	3rd month	6th month	7th month	9th month	13th month	15th month	3rd year
Agent	Methylprednisolone (3 mg/kg/day)	Prednisolone (2 mg/kg/day)	Prednisolone (2 mg/kg/day)	Prednisolone (1 mg/kg/day)	—	Prednisolone (2 mg/kg/day)	Prednisolone (2 mg/kg/day)	—	—	—
	Transfusion						Azathioprine (2 mg/kg/day)	Azathioprine (3 mg/kg/day)	—	—
								Rituximab		
Hb	5.1	9.2	11.5	12.3	12.8	7.3	11.6	9.8	12.9	13.2
Rets	220	120	92	79	75	186	95	115	83	62
DAT	+++ IgG	+++ IgG	+++ IgG	+++ IgG	+++ IgG	+++ IgG	+++ IgG	+++ IgG	+++ IgG	Negative
LDH	1540	720	523	310	285	1230	310	370	290	250
TBL	3.8	1.9	1.2	0.9	0.9	2.9	1.0	1.2	0.9	0.8

Hb (g/dl): hemoglobin; Rets (x 10^3^/*μ*L): reticulocytes; DAT: direct antiglobulin test; LDH (U/L): lactate dehydrogenase; TBL (mg/dl): total bilirubin.

**Table 2:** Basic data from case reports and studies regarding the use of rituximab in children with primary or secondary autoimmune hemolytic anemia.

First author/year	Number of patients	Age	Primary/secondary	Response	Time to response	Duration of response/follow-up	IVIG	Complications
Quartier et al. [8]	6	7–35 mo	5/1	CR	4 min	15–22 min	Y	Pyelonephritis, febrile bronchitis
Zecca et al. [7]	1	18 mo	0/1	CR	2 w	5 min	Y	—
Motto et al. [31]	4	3.5–15 y	3/1	CR	NM	NM	Y	*Pneumocystis carinii* pneumonia, varicella pneumonia
McMahon et al. [32]	1	16 y	1/0	CR	4 w	19 mo	N	—
Hongeng et al. [33]	1	37 mo	0/1	CR	2 w	3 mo	Y	—
Kerridge et al. [34]	1	8 y	1/0	CR	3 w	14 mo	N	—
Zecca et al. [35]	15	0.3–13, 8 y	11/4	13 CR, 2 NR	5–72 d	7.3–27.6 mo	Y	Varicella infection
Gottardo et al. [36]	1	2 y	1/0	CR	1 w	19 mo	Y	—
Wakim et al. [37]	1	6 y	0/1	CR	1 min	16 mo	Y	—
Raj et al. [38]	1	14 y	0/1	CR	3 w	22 mo	N	—
van Daalen et al. [39]	4	3–16 y	4/0	3 CR, 1 NR	NM	NM	Y	—
Bonduel et al. [40]	1	13 mo	0/1	CR	4 w	5 mo	Y	—
Silvana et al. [41]	1	23 mo	0/1	CR	18 d	7 mo	Y	—
Kim et al. [42]	4	3–16 mo	0/4	CR	1–5 w	NM	Y	—
Schappi et al. [43]	1	16 mo	0/1	CR	10 w	18 mo	N	—
Rao et al. [10]	8	5–17 y	6/2	4 CR, 2 PR, 2 NR	NM	3–30 mo	Y	Mild infusion reactions
Beretta et al. [44]	1	8 mo	1/0	PR	2 relapses	7 mo	N	—
Simms-Waldrip et al. [45]	1	9 y	0/1	CR	3 w	4 mo	N	—
Svahn et al. [11]	4	1–9 mo	2/2	3 CR, 1 PR	1–8 min	21–80 mo	Y	—
Lucchini et al. [46]	1	3 y	1/0	CR	2 w	12 mo	N	—
Rao et al. [30]	3	1–9 y	0/3	NR	Relapses	-	Y	Neutropenia
Haller et al. [47]	1	4 mo	0/1	CR	3 w	12 mo	Y	—
Ansari et al. [12]	5	3–14 y	5/0	CR	NM	8–30 mo	N	Seizures during infusion, meningitis
van der Linde et al. [48]	1	2 y	1/0	PR	1 relapse, 11 min	6 y	N	—
Gupta et al. [13]	2	0.5/4 y	2/0	CR/PR	3 w	12 mo	N	—
Kuzmanovic and Jurisic [49]	1	5 mo	1/0	CR	3 w	12 mo	Y	—
Moriya et al. [50]	1	3 mo	1/0	CR	4 w	30 mo	N	—
O'Connell et al. [51]	1	4 y	0/1	PR	NM	NM	N	—
Makadia et al. [52]	1	4 mo	1/0	CR	4 w	6 mo	N	*Pneumocystis carinii* pneumonia
Faraci et al. [53]	7	2–6 mo	0/7	CR	1–3 m	3.5–140 mo	NM	—
Dev et al. [54]	1	9 y	1/0	CR	NM	12 mo	N	Respiratory tract infections
Sankaran et al. [4]	5	NM	NM	2 CR, 1 PR, 2 NR	NM	NM	NM	NM
Ducassou et al. [15]	61	3.3–14.9 y	46/15	40 CR, 6 PR, 15 NR	1–3 min	2.5–7.8 y	13 cases	3 allergic reactions, 1 neutropenia
Ajmi et al. [14]	1	10 y	1/0	CR	3 w	4 mo	N	—
Gonzalez-Vicent et al. [55]	40	NM	0/40	CR or PR	NM	NM	NM	—
